# Plant and pathogen nutrient acquisition strategies

**DOI:** 10.3389/fpls.2015.00750

**Published:** 2015-09-17

**Authors:** Urooj Fatima, Muthappa Senthil-Kumar

**Affiliations:** National Institute of Plant Genome ResearchNew Delhi, India

**Keywords:** apoplast, bacterial pathogen, non-host resistance, nutrients, phloem, phyllosphere, type III secretion system, xylem

## Abstract

Nutrients are indispensable elements required for the growth of all living organisms including plants and pathogens. Phyllosphere, rhizosphere, apoplast, phloem, xylem, and cell organelles are the nutrient niches in plants that are the target of bacterial pathogens. Depending upon nutrients availability, the pathogen adapts various acquisition strategies and inhabits the specific niche. In this review, we discuss the nutrient composition of different niches in plants, the mechanisms involved in the recognition of nutrient niche and the sophisticated strategies used by the bacterial pathogens for acquiring nutrients. We provide insight into various nutrient acquisition strategies used by necrotrophic, biotrophic, and hemibiotrophic bacteria. Specifically we discuss both modulation of bacterial machinery and manipulation of host machinery. In addition, we highlight the current status of our understanding about the nutrient acquisition strategies used by bacterial pathogens, namely targeting the sugar transporters that are dedicated for the plant’s growth and development. Bacterial strategies for altering the plant cell membrane permeability to enhance the release of nutrients are also enumerated along with in-depth analysis of molecular mechanisms behind these strategies. The information presented in this review will be useful to understand the plant–pathogen interaction in nutrient perspective.

## Introduction

**FIGURE 1:**
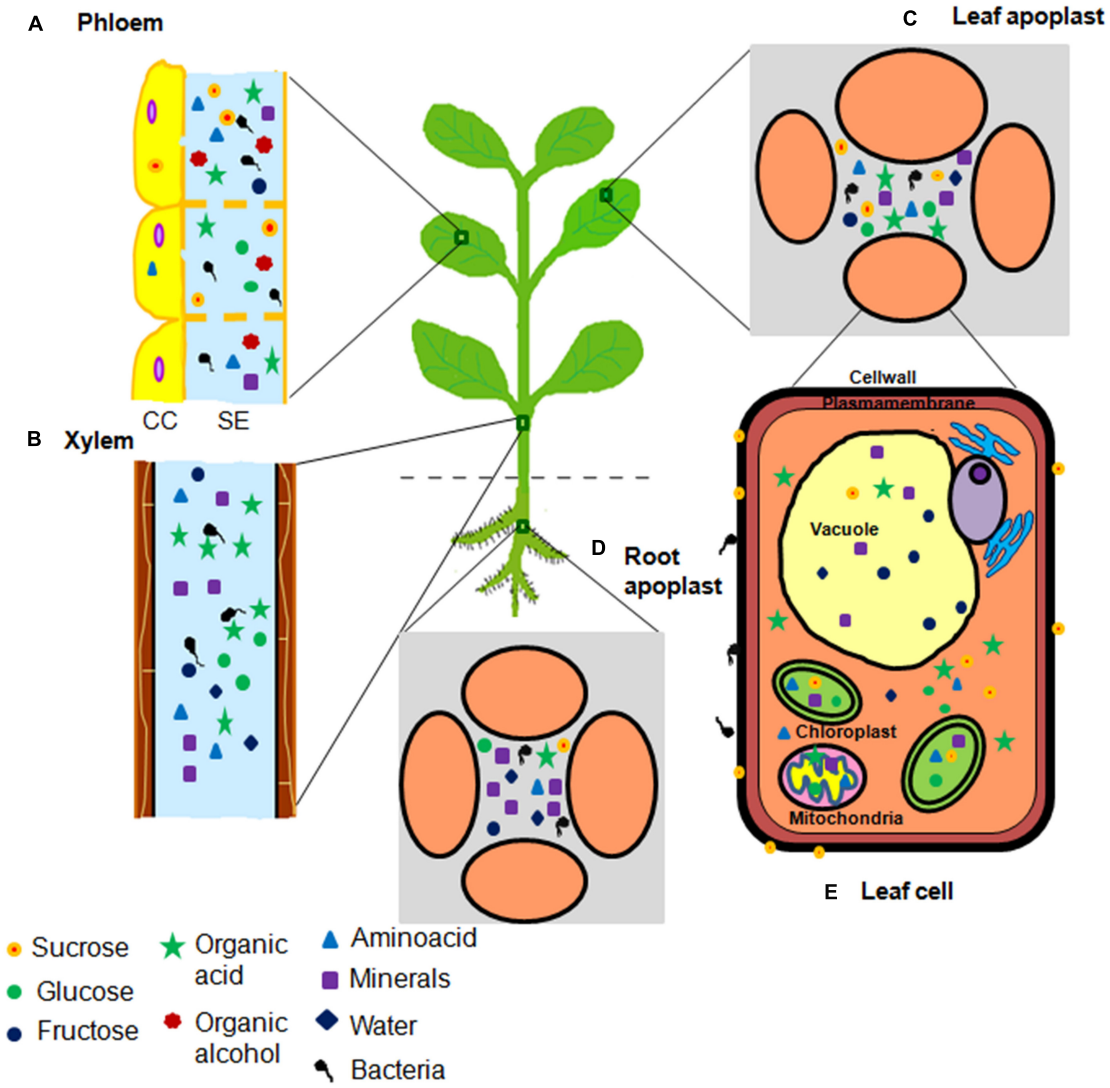
**Illustration of nutrient niches in plants accessed by bacterial pathogens.** Plants predominantly have five nutrient niches namely **(A)** phloem, **(B)** xylem, **(C)** leaf apoplast, **(D)** root apoplast, and **(E)** cell organelles. These niches serve as nutrient reservoirs for invading bacterial pathogen(s). Nutrient content varies in these niches ranging from different types of minerals and carbon sources including sugars, amino acids, organic acids, and organic alcohols. Circles indicate sugars. Triangles indicate amino acids. Square indicates minerals. Five point star indicates organic acids. Seven point star indicates organic alcohols. Diamonds indicate water. Number of symbols indicate the abundance of nutrients.

Pathogenic bacteria infect host plants to acquire nutrients. Initially, bacterial pathogens colonize the plant surfaces namely, the phyllosphere and the rhizosphere and obtain nutrients. Later, majority of them gain access to the interior portions of plant tissues including the vascular elements and the intercellular spaces to obtain more nutrients and to avoid harsh and fluctuating environmental conditions (Beattie and Lindow, 1995, 1999; Melotto et al., 2008; Vorholt, 2012; Griffin and Carson, 2015). The nutrients and favorable environmental conditions inside the plants help pathogens to grow and multiply at high densities, eventually causing serious diseases. Bacterial pathogens enter plants through pre-existing openings such as stomata (for example, *Pseudomonas syringae* pv. *tomato*, causal agent of bacterial speck of tomato; Melotto et al., 2008; Griffin and Carson, 2015), nectarthodes (for example, *Erwinia amylovora*, causal agent of fire blight of apple; Melotto et al., 2008; Griffin and Carson, 2015), hydathodes (for example, *Xanthomonas oryzae* pv. *oryzae*, causal agent of bacterial blight of rice, Nino-Liu et al., 2006; Griffin and Carson, 2015), and lenticels (for example, *E. carotovora* pv. *atroseptica*, causal agent of blackleg of potato; Adams, 1975). They also enter through abrasions on leaf, stem or root and are transmitted through sucking insects (for example, *Xylella fastidiosa*, causal agent of Pierce’s disease of grapevine and variegated chlorosis of citrus) feeding on vascular elements (Purcell and Hopkins, 1996; Griffin and Carson, 2015). Once inside the plant, different pathogenic bacteria inhabit different parts of plant tissues termed in this review as a ‘niche’. Plant niches that harbor pathogens can be defined on the basis of distinct anatomical features of plant tissue, variation in nutrient contents and the difference in their access to pathogens. Based on this, we classified the nutrient niches in six different types namely, phyllosphere, rhizosphere, apoplast, xylem, phloem, and cell organelles (**Figure 1**). Bacterial pathogens are mainly limited to the apoplast, but some can inhabit xylem or phloem cells to obtain nutrients (Bove and Garnier, 2002; Rico and Preston, 2008). Sugars and amino acids are predominantly present in the phloem and the leaf apoplast whereas, mineral nutrients and water are abundant in the xylem and the root apoplast (Myburg et al., 2001; Dinant et al., 2010).

Biotrophic pathogens require living host tissues that ensure prolonged supply of the carbon and other nutrients to them. Necrotrophic pathogens cause host cell death and acquire nutrients released from dead cells. Hemibiotrophic pathogens obtain nutrients from the host cell by exhibiting the biotrophic phase in early stages of the infection process and then switching to the necrotrophic mode during later stages of their life cycle (Glazebrook, 2005). These three types of pathogens utilize different strategies to acquire nutrients. They can either modulate their own metabolism or manipulate the plant cell machinery to acquire nutrients. For instance, bacterial pathogens use transporters for the uptake of desirable and readily available nutrients (Delmotte et al., 2009). Further, they can also take up less preferred nutrients as such or modify it to a desirable form using extracellular enzymes (Jacobs et al., 2012). Biotrophs and hemibiotrophs use type-III secretion system (TTSS) to inject the effector molecules that target different components of the host cell machinery and modulate the plant metabolism for releasing nutrients (Gohre and Robatzek, 2008; Cunnac et al., 2009). For example, some effector molecules facilitate the sugar eﬄux by inducing the expression of genes encoding sugar transporters or genes involved in changing the membrane permeability characteristics of host cell (Chen et al., 2010; Wang et al., 2012). However, necrotrophs secrete cell wall-degrading enzymes that break down the components of host cell wall leading to the release of nutrients (Barras et al., 1994). In this review, we provide an overview of different plant niches and the critical nutrients that are utilized by the bacterial pathogens for their survival (Supplementary Table S1). We also enumerate different types of nutrient acquisition strategies used by pathogenic bacteria for their multiplication in the host plants.

## Nutrient Niches in Plants and their Constituents

Phyllosphere and rhizosphere are important plant surface nutrient niches for bacterial inhabitation, where the bacteria can survive and sometimes grow as epiphyte (Vorholt, 2012; Mendes et al., 2013; Griffin and Carson, 2015). However, the phyllosphere is a hostile environment for most of the bacteria due to many surface-associated stresses such as inconsistency in temperature and relative humidity, as well as direct exposure to ultraviolet rays (Beattie and Lindow, 1995; Lindow and Brandl, 2003; Vorholt, 2012; Griffin and Carson, 2015). Bacteria also face nutrient limitation because waxy cuticle of leaves restricts diffusion of nutrients from the plant interior to the phyllosphere (Lindow and Brandl, 2003; Xiao et al., 2004) and no active transport of nutrients is reported from the cellular interior to the phyllosphere (Derridj, 1996; Riederer, 2008). Carbon sources including sugars, amino acids, and organic acids are the determinants of bacterial colonization (Mercier and Lindow, 2000; Vorholt, 2012; Griffin and Carson, 2015) and these are leached through stomata, hydathodes, and glandular trichomes or wounds (Leveau, 2006). Besides, few minerals like phosphate, calcium, magnesium, potassium, sodium, and manganese also leach out passively from the leaf interior to the surfaces (Tukey, 1970). Glucose, fructose, and sucrose are predominant carbon sources in the phyllosphere followed by amino acids like γ-amino butyric acid (GABA) and organic acids (Supplementary Table S2; Tukey, 1970; Fiala et al., 1990; Weibull et al., 1990; Lindow and Brandl, 2003; Vorholt, 2012). However, the distribution of nutrients in phyllosphere is not homogeneous. At few places, they are abundant due to localized accumulation at the site of leakage (Derridj, 1996). Such places can serve as a microhabitat for bacteria (Vorholt, 2012; Griffin and Carson, 2015). Bacterial pathogens can actively consume the carbon sources present in the phyllosphere (Delmotte et al., 2009). There is a correlation between carbon source availability in the phyllosphere and its utilization profile of pathogen. For example, the *in vitro* carbon source utilization profile of *P. syringae* pv. *tomato* indicates the utilization of those carbon sources which are predominantly present in the phyllosphere such as glucose, sucrose, fructose, organic acids including succinic acid, malic acid, and citric acid and amino acids including alanine, serine, asparagine, glutamic acid, aspartic acid, and GABA (Tukey, 1970; Fiala et al., 1990; Weibull et al., 1990; Ji and Wilson, 2002; Innerebner et al., 2011; Griffin and Carson, 2015). In fact, the pattern of their colonization is found to be similar to that of the nutrient distribution (Lindow and Brandl, 2003; Monier and Lindow, 2004). In order to know the location and distribution of nutrients in the phyllosphere, green fluorescent protein (GFP)-based bacterial sugar-responsive biosensors were used. Bean plants were inoculated with *Erwinia herbicola* 299R (*Eh*299R), harboring plasmid carrying the promoter region of the fructose B (*fruB*) operon from *Escherichia coli* fused with GFP-encoding gene. *Eh*299R fructose-responsive biosensors demonstrated that the distribution of *E. herbicola* correlates with the availability of fructose in the phyllosphere (Leveau and Lindow, 2001; Lindow and Brandl, 2003; Griffin and Carson, 2015). Similarly, in another study, *E. herbicola* harboring plasmid that carried sucrose-responsive promoter fused with *GFP* gene was used to demonstrate the spatially variable distribution of sucrose on the bean leaves (Miller et al., 2001; Lindow and Brandl, 2003; Griffin and Carson, 2015). These studies depicted the location and heterogeneous distribution of nutrients in the phyllosphere. Such studies also suggest that the availability of nutritional resources determine the fate of bacterial colonization in the phyllosphere.

The rhizosphere is another nutrient niche that harbors many non-pathogenic and pathogenic bacterial communities (Raaijmakers et al., 2009; Mendes et al., 2013). Beneficial rhizosphere bacteria are well studied (Mendes et al., 2013). However, only few bacterial pathogens are known that infect the plants through roots. They include *Ralstonia solanacearum* (causal agent of bacterial wilt of tomato), *Agrobacterium tumefaciens* (causal agent of crown gall tumor), *Dickeya dadantii* (causal agent of soft rot diseases of several crops) and *Dickeya solani* (causal agent of soft rot diseases of potato), *Pectobacterium carotovorum* (causal agent of soft rot diseases of several crops), and *Pectobacterium atrosepticum* (causal agent of black leg diseases of potato) and these were ranked among the top 10 plant pathogenic bacteria (Mansfield et al., 2012; Mendes et al., 2013). Rhizosphere colonization is the primary step involved in the pathogenesis of bacterial pathogen. Nutrient sources available in rhizosphere can influence the bacterial population and play an important role in the niche colonization (Somers et al., 2004; Dennis et al., 2010; Griffin and Carson, 2015). The rhizosphere is enriched with large quantities of sugars, amino acids, organic acids, and minerals in the form of root exudates (Supplementary Table S2). The most abundant nutrients available in the root exudates determine the ability of bacteria to colonize the rhizosphere. For example, organic acids such as citric acid, malic acid, lactic acid, and succinic acid are the major nutrients present in tomato root at amount five times higher than that of the sugars (Lugtenberg et al., 1999). *Psuedomonas fluorescens* mutants impaired in their ability to utilize organic acids were not able to colonize tomato root and showed reduced growth in rhizosphere. On the other hand, a mutant impaired in their ability to utilize sugars normally colonized the rhizosphere (Lugtenberg et al., 1999, 2001; Somers et al., 2004). This suggests the direct correlation between nutrient abundance in the niche and nutrient utilization ability of the bacteria.

Apoplast provides conducive environment and more protection for host pathogens from many surface-associated responses (Beattie and Lindow, 1995; Hirano and Upper, 2000). Therefore, it is the major site for colonization by bacterial pathogens (Sattelmacher and Horst, 2007; Rico and Preston, 2008; Rico et al., 2009). Nutrients present in the apoplast mainly flow from the cytosol of surrounding cells (Sattelmacher, 2001). Further, an active eﬄux is also reported. For example, during phloem loading in *Arabidopsis thaliana*, sucrose is actively exported from the phloem parenchyma cells into the apoplasm by AtSWEET11 (Sugars Will Eventually Be Exported Transporter) and AtSWEET12 sucrose eﬄux transporters (Baker et al., 2012; Chen et al., 2012; Eom et al., 2015). Major sources of carbon nutrients identified in the leaf apoplast include GABA, aspartate, glutamate, fructose, glucose, citrate, succinate, malate, and malonate (Supplementary Tables S1, S2, and S3; Rico and Preston, 2008). However, most of the apoplastic nutrients are bound in the region of cell wall or stored in the vacuole present inside the cell. Thus, the apoplastic nutrients are not easily available to pathogenic bacteria as reported for *P. syringae* pv. *tomato* (Melotto et al., 2008; Rico and Preston, 2008). In addition, apoplast has less water availability compared to the phyllosphere (Yu et al., 2013) and this is one of the major factors that determine the colonization of a bacterial pathogen (Wright and Beattie, 2004; Beattie, 2011). Owing to these conditions, host pathogen has to employ strategies to release nutrients and water into the apoplast. Apart from this, pH is another determining factor for bacterial multiplication. Low pH restricts the multiplication of phyto-pathogenic bacteria (Nakka et al., 2010). For example, *P. syringae* pv. *syringae* experience slight acidic environment in the apoplast, therefore, during compatible interactions they promote apoplast alkalinization for their favorable multiplication (Atkinson and Baker, 1987, 1989; Grignon and Sentenac, 1991). Also *P. syringae* pv. *syringae* produce syringomycin and disrupts H^+^ channel to alkalinize the apoplast (Hutchison et al., 1995). Alkalinization increases the availability of nutrients to the bacteria. Studies suggest that pathogens have evolved nutrient acquisition strategies based on nutrient abundance in the host plant. Bacterial pathogens express specific nutrient utilization pathways to utilize the most abundant nutrient present in apoplast. For example, *P. syringae* pv. *tomato* inhabiting tomato (*Solanum lycopersicum*) apoplast uses GABA, the most abundant amino acid present in tomato apoplast (Rico and Preston, 2008). Accordingly, the genome of *P. syringae* pv. *tomato* has evolved in such a way that it contains three copies of two genes needed for GABA metabolism, whereas other *Pseudomonas* strains contain a single copy (Rico and Preston, 2008). This study indicates the relation between the nutrient utilization abilities of bacterial pathogens and the corresponding nutrient availability in host plants. This line of research is expected to reveal in-depth information on evolution of the bacterial nutrient acquisition strategies in accordance with nutrient abundance. This necessitates more systematic studies for deeper mechanistic understanding in this direction.

Vascular tissue comprising xylem and phloem serve as another nutrient niche for pathogens. Phloem consists of sieve tube elements associated with companion cells and functions in the transport of sugar from source to sink tissues (van Bel and Knoblauch, 2000). Sucrose is loaded from the apoplast into the companion cells and then it flows into the phloem sap of sieve tube elements (Giaquinta, 1983; Riesmeier et al., 1994). The nutrient-rich phloem sap includes sugars, organic acids, amino acids, sugar alcohols, and some minerals (Supplementary Tables S2 and S3). For example, metabolomic analysis of squash (*Cucurbita maxima*) phloem sap revealed sucrose, glucose, and fructose as the predominant source of carbon, followed by organic acids including citrate and malate (Fiehn, 2003). Similarly, phloem of the plants belonging to the family Rosaceae, contains sugar alcohol sorbitol as a major carbon source instead of sucrose which is otherwise predominant in other plants. Pathogenic bacteria such as *E. amylovora* that infect Rosaceae family plants uses sorbitol as preferred carbon source (Aldridge et al., 1997). This supports the notion that the type of nutrient present in a plant determines the type of pathogen it harbors. Nevertheless, host and pathogen are involved in a process of co-evolution that happens at all levels, not only at the level of nutrient acquisition. Spiroplasmas and phytoplasmas are strictly limited to the phloem, whereas some strains of *Pseudomonas* such as *P. syringae* pv. *actinidiae* (causal agent of bacterial canker of kiwifruit) and *P. syringae* pv. *aesculi* (causal agent of bleeding canker of horse chestnut) inhabit phloem as well as other nutrient niches (Supplementary Table S2; Bove and Garnier, 2002; De Keijzer et al., 2012; Renzi et al., 2012).

Xylem is mainly composed of dead and lignified cells and, therefore, xylem sap has cell wall breakdown products (Pieretti et al., 2012). Compared to other nutrient niches xylem contains the lowest concentration of organic carbon (Supplementary Table S3). Only traces of diverse organic acids, amino acids and few sugars present in xylem serve as nutrient source for pathogenic bacteria (Supplementary Table S2; Press and Whittaker, 1993). For example, major organic acids found in the sugar beet xylem sap include malate, citrate, and succinate (Lopez-Millan et al., 2000). Similarly, tomato xylem sap contains small amount of glucose and fructose as well as low concentrations of glutamine followed by asparagine, GABA and other amino acids (Supplementary Table S3; Zuluaga et al., 2013). Xylem also contains monomeric inorganic ions that are essential for the bacteria. Estimation of xylem sap content from different plant species showed potassium as the highly abundant mineral nutrient followed by nitrate and chloride (http://plantsinaction.science.uq.edu.au/edition1, Supplementary Table S3). *Xanthomonas albilineans* (causal agent of sugarcane leaf scald) and *Xylella fastidiosa* are xylem limited bacteria (Bove and Garnier, 2002; Pieretti et al., 2012). Some bacteria belonging to genus *Erwinia, Pseudomonas, Ralstonia*, and *Xanthomonas* also inhabit the xylem apart from other niches (Supplementary Table S2; Bove and Garnier, 2002).

Plant cell organelles are another important niche for many necrotrophic pathogens (Alfano and Collmer, 1996). It serves as a prime nutrient source for other nutrient niches like apoplast (Sattelmacher, 2001). Bacterial pathogens use various nutrient acquisition strategies to drive nutrients from the cytosol into the apoplast (Chen et al., 2012; Chen, 2014). Cytosol contains glucose, fructose and sucrose as major sugars, as well as organic acids, amino acids, and some minerals. The vacuole is a long-term and large store house for soluble sugars. Fructose is a predominant form of stored sugar found in the vacuole of barely (*Horduem vulgare*) and *Arabidopsis* (Martinoia et al., 1987; Guo et al., 2014).

## Recognition of Nutrient Niche and Regulation of Pathogenicity Factors in Response to Nutrient Availability

Quorum sensing is efficient way of regulating the expression of specific bacterial genes in response to bacterial cell density. This is based on the constant production and detection of signaling molecules known as auto inducers (Helman and Chernin, 2014). *N*-acylhomoserine lactones (AHLs) produced in *Erwinia* sp and *Pseudomonas* sp are common group of autoinducer signals. Other examples of autoinducer signals are 3-hydroxy palmitic acid methyl ester (3-OH PAME) in *R. solanacearum* and α, β unsaturated fatty acid (*cis*-11-methyl-2-dodecenoic acid) in *X. campestris* (Gonzalez and Keshavan, 2006). Quorum sensing allows the co-ordination of bacterial population to enhance access to the nutrient-rich niches (Williams, 2007). Quorum sensing plays a role in the formation of biofilms (O’Toole et al., 2000; Jayaraman and Wood, 2008). Biofilms are highly organized structures, composed of bacterial communities enmeshed in the polysaccharide matrix that protects the bacterial communities from water loss (Flemming and Wingender, 2010). It is known that bacteria initiate biofilm formation in response to nutrient availability (O’Toole et al., 2000; Shrout et al., 2006; De Kievit, 2009). Based on these facts, we predict that bacteria sense the nutritional content of the niches through quorum sensing and trigger the formation of biofilms as a mechanism for nutrient acquisition and desiccation prevention. Apart from this, chemotaxis and motility also play role in recognition of nutrient niches and its colonization.

Further, we suggest that after sensing the scarcity of nutrients through quorum sensing, bacterial pathogens regulate the expression of *Hrp* (for hypersensitive reaction and pathogenicity) and other genes that facilitate them to overcome the nutrient limitation by acquisition of more nutrients. *Hrp* gene expression is induced in minimal medium or *in planta* but not in nutrient-rich medium (Alfano and Collmer, 1996; Brito et al., 2002). This suggests that nutrient and environmental signals regulate the *Hrp* gene expression in phytopathogenic bacteria (Alfano and Collmer, 1996). Quorum sensing indirectly plays role in regulating the expression of *Hrp* genes by sensing the nutrient concentration. For example, in *R. solanacearum* HrpG is the response regulator of TTSS activation cascade and HrpB is the transcriptional activator of *Hrp* gene expression. HrpG induces the expression of *HrpB* gene that in turn activates the expression of *Hrp* genes encoding TTSS and effector molecules (Brito et al., 1999; Valls et al., 2006). Quorum sensing also regulate the expression of genes encoding plant cell wall-degrading enzymes in *Pectobacterium atrosepticum* and *Pectobacterium carotovorum* and these enzymes facilitate the release of nutrients by degrading cell wall (Jones et al., 1993; Davidsson et al., 2013).

Similarly, quorum sensing is proposed to act as master regulator for virulence genes that facilitates the transition from biotrophy to necrotrophy in hemibiotrophic bacterial pathogens (Liu et al., 2008; Davidsson et al., 2013). Based on this, we propose a hypothesis that in hemibiotrophic bacterial pathogen, nutrient limitation sensed through quorum sensing plays a role in mediating transition from biotrophy to necrotrophy. During asymptomatic biotrophic phase, when hemibiotrophic bacterial pathogens sense their population density and nutrient scarcity, they switch themselves to the necrotrophic mode of infection. Therefore, during necrotrophic phase, hemibiotrophic bacterial pathogens can grow into large population sizes by obtaining more nutrients through plant tissue degradation (Jones et al., 1993; Liu et al., 2008; Davidsson et al., 2013). Further, effector molecules are known to regulate the switch from biotrophy to necrotrophy in fungus. For example, *Phytophthora infestans*, a hemibiotrophic fungal pathogen secrete SNE1 (secreted effector protein) specifically in biotrophic phase that suppresses necrosis inducing effectors (Lee and Rose, 2010). Such effectors are not yet identified in bacterial pathogens. More systematic studies are needed to relate the role of these effectors in mediating transition from biotrophy to necrotrophy.

## Bacterial Strategies for Nutrient Acquisition From Plants

Necrotrophic bacteria employ cell wall degrading exoenzymes, necrosis inducing proteins and toxins to provoke cell death (Alfano and Collmer, 1996; Glazebrook, 2005; Laluk and Mengiste, 2010). Some plant cell wall-degrading exoenzymes used by the bacteria are pectate lyase, pectin lyase, pectin methylesterase, cellulase, polygalacturanase, and protease. All of these exoenzymes except protease are secreted by type II secretion system (Johnson et al., 2006; Pieretti et al., 2012). These exoenzymes facilitate the bacterial entry in the plant tissues by breaking down the component of host cell wall and expose the nutrients from the cytosol and the various organelles (Alfano and Collmer, 1996; Glazebrook, 2005; Laluk and Mengiste, 2010). Biotrophs and hemibiotrophs obtain nutrients by manipulating the expression of plant genes regulating cell membrane permeability or the genes encoding transporters. Apart from this, some bacteria modulate their own machinery to suitably utilize the available host nutrients. We discussed the strategies used by biotrophs and hemibiotrophs for nutrient acquisition in subsequent section.

## Strategies for Modulation of Pathogen Machinery for Nutrient Acquisition

**FIGURE 2:**
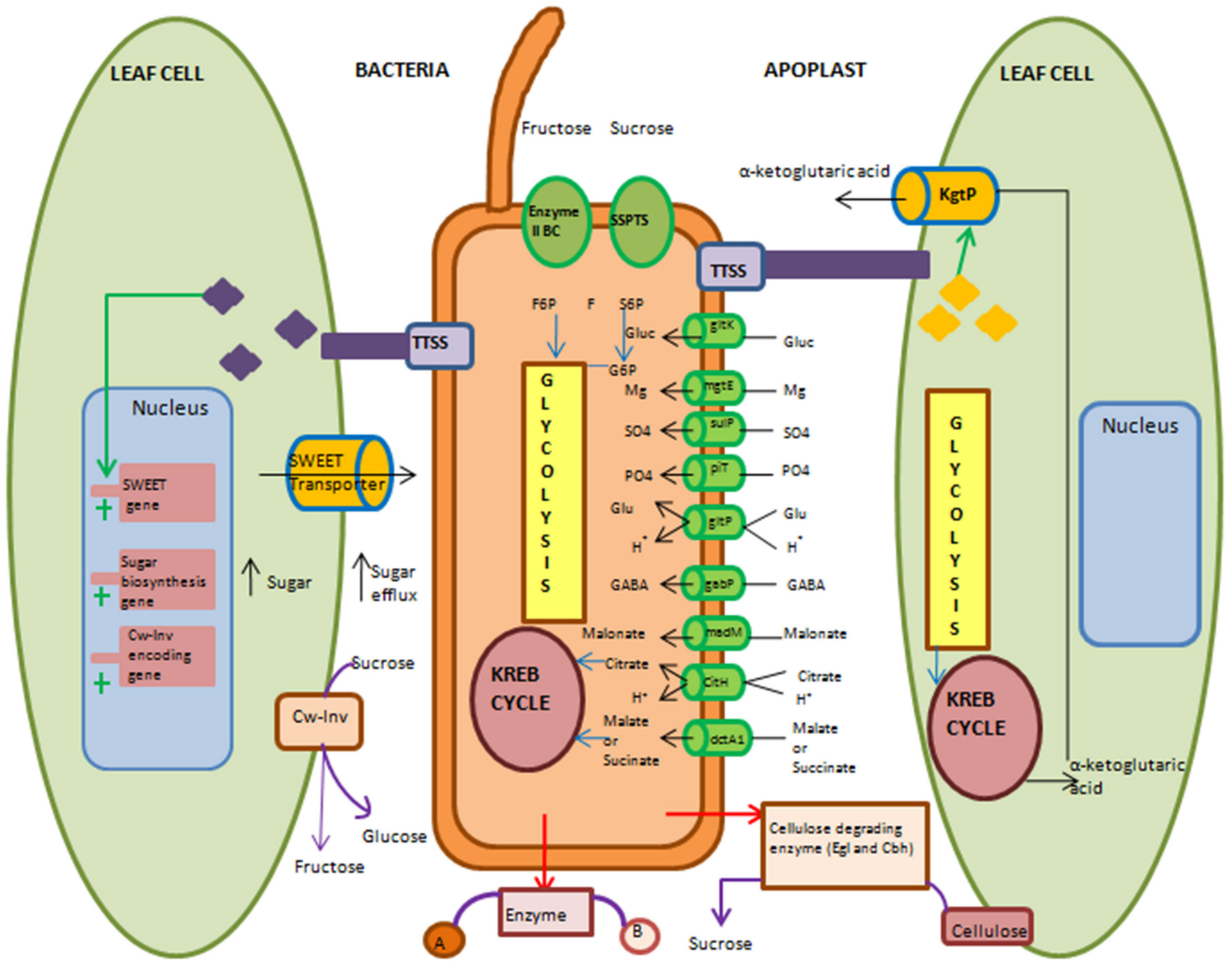
**Overview of molecular and biochemical events used by biotrophic or hemibiotrophic bacteria to acquire nutrients during apoplast colonization.** Pathogenic bacteria use several strategies to acquire nutrients. They can either modulate their own machinery or manipulate plant cell machinery to acquire nutrients. During modulation of their own machinery, bacteria activate various transporters and take up nutrients that are present in apoplast. They can secrete cellulose degrading enzyme to release cell wall-bound nutrients (Cw-Inv). For uptake of less preferred nutrients they use two different ways. First, by secreting enzyme in apoplasm that converts undesirable form of nutrient into desirable form and then take up that desirable form of nutrient by transporter. Second, the uptake of less preferred nutrient by specific transporter and then suitably metabolize its energy. Sucrose specific phosphotransferase system is shown here for uptake of sucrose in the form of sucrose-6-phosphate (S6P) and catabolize it into fructose (F) and glucose-6-phosphate (G6P). Type III secretion system (TTSS) delivered effectors target expression of sugar transporter-encoding genes of host cell. Effector-mediated induction of SWEET (sugar will eventually be exported transporter) transporter for increasing sugar eﬄux in apoplast is shown here. Also induction of sugar biosynthesis genes for high sugar synthesis in cytosol and its movement to apoplast is shown. Expression of host cell wall-invertase-mediated conversion of sucrose into glucose and fructose in apoplast is depicted. α-Ketoglutaric acid transporter (KgtP) secreted by bacterial pathogen though TTSS and its localization in host cell membrane and the eﬄux of α-ketoglutaric acid from host cell into apoplasm is illustrated. Enzyme II BC, Fructose specific phosphotransferase system; SSPTS, Sucrose-specific phosphotransferase system; gltK, glucose ATP-binding cassette transporters (ABC) transporter permease; mgtE, magnesium transporter; fecC, Iron ABC transporter permease; sulP, sulphatepermease; piT, inorganic phosphate transporter; gltP, proton/glutamate symport; gabP, gamma-amino butyric acid (GABA) permease; madM, malonate transporter; citH, citrate/proton symport; dctA1, dicarboxylate transporter; kgtP, ketoglutaric acid transporter; egl, endoglucanase; cbhA, cellobiohydrolase; f6p, fructose-6-phosphate. Black arrow indicates influx or eﬄux of nutrients. Red arrow indicates extracellular enzyme secretion. Violet arrow indicates extracellular enzymatic reaction. Blue arrow indicates entry of nutrient into metabolic pathway. Green arrow indicates delivery through TTSS. Cylinder indicates transporter. Plus sign indicates induction of genes.

The first strategy for obtaining nutrients from niches involves the use of transporters present in the pathogen. Porins present in bacterial membrane allow the passive diffusion of nutrients available in plant niches. These pore-forming proteins are abundant in several species of *Pseudomonas* (Delmotte et al., 2009). Bacterial pathogens also use different types of transporters for the active uptake of nutrients (**Figure 2**). ATP-binding cassette transporters (ABC-transporters) and TonB-dependent transporters (TBDTs) are the important transporters involved in nutrient uptake (Delmotte et al., 2009). For example, ABC-transporters present in *Pseudomonas* species facilitate the uptake of maltose, glucose and sucrose as well as amino acids and minerals (Delmotte et al., 2009). TBDTs are known to be involved in iron or vitamin B12 uptake. These transporters are also shown to facilitate the uptake of carbohydrates present in low amounts on the leaf surfaces (Lindow and Brandl, 2003; Vorholt, 2012). Genomes of several species of *Xanthomonas, Sphingomonas*, and *Pseudomonas* are known to have high representation of *TBDT* genes. For example, the genomes of *X. campestris* pv. *campestris* (causal agent of black rot of crucifers), *P. syringae* pv. *tomato* DC3000 and *P. syringae* pv. *syringae* (causal agent of blight of barely) have 72, 25, and 19 *TBDT* genes, respectively (Blanvillain et al., 2007; Cornelis and Bodilis, 2009). During phyllosphere colonization, *X. campestris* pv. *campestris* encounters nutrient limited environment on the leaf surfaces. Under these conditions, TBDT has been shown to transport sucrose available in the phyllosphere (Blanvillain et al., 2007). Similarly, the genome of *X. albilineans* (causal agent of sugarcane leaf scald) has 35 putative *TBDT* genes. During their xylem colonization, TBDT is involved in the transport of plant cell wall derived nutrients like maltose, xylan, xylose, pectin, polygalacturonate, and arabinose (Blanvillain et al., 2007; Pieretti et al., 2012; De Bernonville et al., 2014). These studies show that bacterial pathogens are adapted to live in nutrient-poor environments by scavenging the plant carbohydrates through TBDTs. Other studies indicate that bacterial pathogens preferentially utilize some carbon sources over others. Therefore, they highly express genes encoding transporters that specifically facilitate the uptake of preferred carbon sources present in the niche. For example, some species of *Pseudomonas* and *Xanthomonas* preferentially utilize dicarboxylates such as malate, citrate and succinate over other carbon sources present in the apoplast. Dicarboxylate transporter (DctA1) of *P. syringae* pv. *tomato* strain facilitates the uptake of TCA cycle intermediates (Mellgren et al., 2009). Similarly, citrate transporter (CitH) of *X. campestris* pv. v*esicatoria* facilitates the citrate uptake and it is important for this bacterial virulence in tomato plants (Tamir-Ariel et al., 2011).

The second strategy employed by the bacterial pathogens involves changes in their metabolism for the better utilization of less preferred nutrients. During non-availability of desirable nutrients, bacterial pathogens utilize less preferred nutrients. *R. solanacearum* colonizes the tomato xylem which contains sucrose but not glucose (Jacobs et al., 2012). However, glucose is a preferred carbon source under *in vitro* conditions for this pathogen (Jacobs et al., 2012). *R. solanacearum* metabolizes sucrose present in the xylem by using sucrose-specific phosphoenol pyruvate-carbohydrate phosphotransferase system (SSPTS) encoded by *scrRABY* gene cluster. SSPTS facilitates the sucrose uptake and convert it into glucose-6-phosphate and fructose-6-phosphate in a two-step process (Jacobs et al., 2012). This strategy not only provides the significant fitness advantage during xylem colonization, but also allows the bacteria to exploit other nutrient sources that are otherwise less preferred by them.

The third strategy involves the release of bound nutrients from host cell wall during nutrient starvation. *Xanthomonas albilineans, Xylella fastidiosa*, and *R. solanacearum* use cellulose-degrading enzymes such as endoglucanase (EGL) and cellobiohydrolase (CBH) that alter plant cell wall components to release sucrose (Jacobs et al., 2012; Pieretti et al., 2012). These enzymes are also required for degradation of the xylem pit membrane and nutrient release. This helps successful colonization of the xylem vessels (Pieretti et al., 2012). Further, plants and bacterial pathogens are coevolving with each other and during this plants can evolve strategies to modulate their nutrient availability to the pathogen. Therefore, we predict that successful colonization of niches depends upon how rapidly and effectively the bacterial pathogens can evolve and adapt various nutrient acquisition strategies, involving modulation of their own machinery. This helps the bacterial pathogens to exhaustively utilize different nutrients that are otherwise less prefered by them or not easily accessible from plants. However, we have little knowledge about role of nutrients in plant–pathogen co-evolution and more specific studies are needed to investigate this interesting point.

## Strategies for Manipulation of Host Machinery

During the apoplast colonization, bacterial pathogens manipulate host cell machinery for nutrient release from the cell into the apoplast. For achieving this, the pathogens belonging to genus *Xanthomonas* and *Pseudomonas* use two different strategies. The first strategy involves the use of TTSS for reprogramming of plant carbon metabolism (Supplementary Table S4). Bacterial pathogens deliver effector molecules to enhance the sugar eﬄux and target SWEET transporters (Chen, 2014; Eom et al., 2015). For example, *X. oryzae* pv. *oryzae* PXO99 (causal agent of bacterial leaf blight of rice) injects effector PthXo1, a transcriptional activator-like (TAL) protein that binds directly to the promoter of gene-encoding SWEET11 transporter and induces its expression (Chen et al., 2010). It increases the eﬄux of glucose from the cytosol that can be acquired by pathogen for their enhanced growth in the apoplast. Similarly, *X. oryzae* pv. *oryzae* PXO99 also secretes effector AvrXa7 that up-regulates the expression of plant gene-encoding SWEET14 transporter (Chen et al., 2010; Eom et al., 2015). Recently, OsSWEET13 a sucrose transporter in rice is also known to be the target of *X. oryzae* pv. *oryzae*. This pathogen induces the expression of *OsSWEET13* gene by using PthXo2 effector. This eventually enhances the release of sucrose from host cell into the apoplast that can be exploited by pathogen as a source of nutrition (Eom et al., 2015; Zhou et al., 2015). During compatible interactions, bacterial pathogens induce the expression of gene-encoding cell wall-bound invertase (*Cw-Inv*). Cw-Inv is an extracellular enzyme that converts the sucrose into glucose and fructose in the apoplast and these serve as nutrients for pathogens (Kocal et al., 2008; Sonnewald et al., 2012). However, early induction of cw-Inv activity and the rapid accumulation of soluble sugars during incompatible interactions can induce defense responses (Scharte et al., 2005; Swarbrick et al., 2006; Kocal et al., 2008; Sonnewald et al., 2012) like callose deposition and phenolic compounds production and establish resistance against the pathogens (Herbers et al., 1996a,b; Scharte et al., 2005). Induction of *PR* 1 (pathogenesis related) and *PR* 3 gene expression are also known to be triggered by hexose signals (Herbers et al., 1996a,b, 2000). Further bacterial pathogens are known to secrete the transport protein via TTSS and facilitate the nutrient export from host cell to the pathogen. For example, in rice *X. oryzae* pv. *oryzae* PXO99 injects ketoglutarate transport protein (KgtP). This protein is localized onto the host cell membrane and facilitates the export of α-ketoglutaric acid from host cell to the pathogen. In rice, α-ketoglutaric acid is synthesized by enzyme, iso-citrate dehydrogenase (IDH). Upon infection in rice, this pathogen indirectly enhances the expression of *OsIDH* gene by exporting α-ketoglutaric acid (Guo et al., 2012). This suggests that KgtP present in pathogen indirectly functions in manipulating the plant cell metabolism.

The second strategy involves the manipulation of host cell membrane permeability characteristics by bacterial pathogens for obtaining the nutrients from host cell (Chen, 2014). For example, stigmasterol content plays important role in determining the plant membrane permeability characteristics (Schuler et al., 1991; Bernsdorff and Winter, 2003). A change in the ratio of stigmasterol and sitosterol content alters the membrane permeability. *Arabidopsis AtCYP710A1* gene-encoding C22-sterol desaturase enzyme catalyzes the conversion of β-sitosterol into stigmasterol and plays an important role in maintaining the membrane permeability characteristics (Schuler et al., 1991; Morikawa et al., 2006). During both PAMP triggered immunity (PTI) and effector triggered immunity (ETI) the expression of *AtCYP710A1* gene is induced. *Atcyp710a1* mutant in *Arabidopsis* showed release of nutrients from cytosol into the apoplast and supported higher multiplication for the bacteria belonging to *Pseudomonas* genus (Wang et al., 2012; Chen, 2014). Consistent with the fact that bacterial effectors modulate host machinery (Cunnac et al., 2009), we propose that pathogens inject effectors into the plant cell that interfere with the expression of stigmasterol biosynthesis genes to enhance the release of nutrients from the cytosol into the apoplast.

## Pathogen Host Specificity and Bacterial Nutrient Acquisition Capability in Relation to Non-host Resistance

Non-host resistance of plants prevents a wide range of pathogen infection (Mysore and Ryu, 2004; Nürnberger and Lipka, 2005; Senthil-Kumar and Mysore, 2013). We propound that the non-availability of nutrients in plant and the lack of nutrient acquisition ability are the two considerable factors among several others that dictate the failure of non-host pathogen to infect the non-host plant. Sometimes, nutrients available at particular niche in non-host plant do not match with the nutrients required by the pathogen. Further, less- or non-availability of desirable nutrients restricts the non-host pathogen infection. Many nutrients are sequestered (Melotto et al., 2008; Expert et al., 2012) and not easily accessible by non-host pathogens. Another possible reason could be the lack of nutrient acquisition strategies such that non-host pathogens are not able to modulate their own metabolism and incapable of reprogramming the host cell machinery for acquiring nutrients. On the contrary, the host pathogens adapt various strategies to gain access to these nutrients present in the niches. Taken together, we suggest that nutrient availability or its limitation to pathogens due to plant defense is one of the reasons for non-host resistance (Senthil-Kumar and Mysore, 2013). This is supported by a recent study involving *SQS*-encoding squalene synthase and *SMT2*-encoding sterol methyltransferase 2 that participate in sterol biosynthesis that determines the membrane permeability (Supplementary Table S5). Due to increases in membrane permeability of *NbSQS*-silenced *Nicotiana benthamiana* and *atsmt*-2 *Arabidopsis* mutant plants, the nutrient release in the apoplast increased (Wang et al., 2012). Therefore, upon inoculation with non-host pathogens these plants were compromised in non-host resistance (Senthil-Kumar and Mysore, 2013). Notably, the wild type plants were resistant to these pathogens. This finding implicates that restriction of nutrient availability to invading pathogens can impart non-host resistance. Currently, only this study demonstrates the significance of nutrient availability in non-host resistance. Further, more systematic studies are needed to support this view.

## Conclusions and Future Perspectives

Plant nutrient niches are widely and differentially targeted by the bacterial pathogens and availability of nutrients plays a major role in determining the ability of pathogens to grow and multiply in these niches. Wide variability exists in the nutrient content of different nutrient niches. This indicates that bacterial pathogens have to cope with the inconsistent environment for which they modulate their metabolism in accordance with the nutrient content of different nutrient niches. Bacterial pathogens adapt their metabolic machinery in such a way that they preferentially use the most abundant nutrient sources of particular niche. Notably, different species of bacterial pathogens preferentially utilize some nutrient sources over others. During apoplast colonization, bacterial pathogens secrete effector molecules that target different components of plant cell machinery which enhance the availability of nutrients. Xylem-limited bacterial pathogens possess novel strategies to live in nutrient-poor and cloistered environment.

To date, the role of nutrient availability in biotrophy to necrotrophy transition in hemibiotrophic bacterial pathogens is not well understood. This is one of the interesting future research area. Further, nutrient acquisition strategies used by bacterial pathogens are not well understood. Thus, future studies need to be focussed on the identification of more repertoires of bacterial effectors and their target genes in plant cell that are manipulated to facilitate the nutrient eﬄux for pathogen nutrition. It will be advantageous to mutate effector-binding site of the target gene that prevent effector binding and thereby prevents the pathogen from manipulating nutrient eﬄux system. Recently, transcription activator-like effector nucleases (TALEN)-based promoter editing of *OsSWEET14* gene was done to disrupt TAL-effector binding site and this provided disease resistance in rice against *X. oryzae* pv. *oryzae* (Li et al., 2012; Eom et al., 2015). Further, using host-induced gene silencing (HIGS), plants resistant to a fungal pathogens were developed (Nowara et al., 2010). In the future, HIGS can be used as a powerful approach for silencing the genes involved in metabolism, growth and virulence of invading bacterial pathogens. Another interesting area of research in the near future is to understand non-host resistance strategies used by plants for limiting the nutrient availability to non-host pathogens.

## Conflict of Interest Statement

The authors declare that the research was conducted in the absence of any commercial or financial relationships that could be construed as a potential conflict of interest.
