# Supplementary material for: Plant and pathogen nutrient acquisition strategies
Source: Front Plant Sci. 2015 Sep 17;6:750. doi: 10.3389/fpls.2015.00750 (PMC4585253; doi:10.3389/fpls.2015.00750)
Supplement: Supplementary file 1 [file Table_1.DOCX]

**Supplementary table S1.** Economically important bacterial phytopathogens, their colonizing niche and the critical nutrients required for their survival*

| SI. No | Name of bacterial pathogen | Name of disease caused | Name of nutrient niche colonized | Name of nutrients critically needed ^# | Mode of nutrient utilization^^ | Reference |
| --- | --- | --- | --- | --- | --- | --- |
| 1. | *Pseudomonas syringae* pv*. tomato* DC3000 | Bacterial speck of tomato | Phyllosphere, Apoplast | Succinate  Citrate Malate  Fructose Glucose  GABA  Glutamate  Aspartate  Sucrose  Alanine  Asparagine  Serine | ABC transporter;  DctA transporter  gabP | Ji and Wilson(2002); Mellgren *et al*. (2009); Rico and Preston (2008); Vorholt (2012) |
| 2. | *Ralstonia solanacearum* | Bacterial wilt disease of tomato | Xylem | Glucose  Fructose  GABA  Glutamine Succinate  Citrate  Aspartate  Glutamate  Asparagine  Proline | EII BC PTS, gabP | Yao and Allen (2006); Zuluaga *et al*. (2013) |
| 3. | *Xanthomonas campestris* pv*. campestris* | Black rot disease of crucifer | Apoplast | Succinate Fumarate  Malate  Glucose  Sucrose  Fructose  Mannitol  Glutamate  Aspartate Alanine | ABC transporter;  TBDTs transporter | Jiang *et al.* (2013); Tang *et al.* (2005) |
| 4. | *Xanthomonas campestris* pv*.vesicatoria* | Bacterial leaf spot on pepper and tomato | Apoplast | Succinate  Glutamate  Alanine  Aspartate  Arginine Citrate  Malate Glucose  Fructose  Sucrose  Proline | CitH;  ABC transporter;  TBDTs transporter | Shenge *et al*. (2006); Stoyanova *et al.* (2014); Tamir-Ariel *et al*. (2007) |
| 5. | *Erwinia amylovora* | Fire blight of apple | Phyllosphere, Apoplast, Xylem, Phloem | Sorbitol  Fructose  Glucose  Sucrose  Mannitol  Succinate  Glutamate Aspartate | Sorbitol EII PTS system | Aldrige *et* al. (1997); Atanasova *et al.* (2005) |

* indicates information presented here is regarding few economically important pathogens. ^ indicates only those nutrients are listed here which are available in respective niches and critically needed by bacterial pathogens. # indicates that nutrients are arranged in the order of preference given by bacterial pathogens. ^^ indicates important transporters are listed here.

ABC transporter, ATP-binding cassette transporter; DctA, Dicarboxylate transporter A; EII BC PTS, Enzyme II phosphotransferase system; gabP, Gamma amino butyric acid permease; CitH, citrate/proton symport; TBDTs transporter, TonB-dependent transporters.

**References:**

Aldridge, P., Metzger, M., & Geider, K. (1997). Genetics of sorbitol metabolism in *Erwinia amylovora* and its influence on bacterial virulence. *Molecular General Genetics*, 256, 611-619.

Atanasova, I., Kabadjova, P., Bogatzevska, N., & Moncheva, P. (2005). New host plants of *Erwinia amylovora* in Bulgaria. *Zeitschrift für Naturforschung C*,*60*(11-12), 893-898.

Ji, P., & Wilson, M. (2002). Assessment of the importance of similarity in carbon source utilization profiles between the biological control agent and the pathogen in biological control of bacterial speck of tomato. *Applied and environmental microbiology*, 68(9), 4383-4389.

Jiang, G. F., Jiang, B. L., Yang, M., Liu, S., Liu, J., Liang, X. X., & Tang, J. L. (2013). Establishment of an inducing medium for type III effector secretion in *Xanthomonas campestris* pv. campestris. *Brazilian Journal of Microbiology*,*44*(3), 945-952.

Mellgren E. M., Kloek A. P., & Kunkel B. N. (2009). Mqo, a tricarboxylic acid cycle enzyme, is required for virulence of *Pseudomonas syringae* pv. *tomato* strain DC3000 on *Arabidopsis thaliana*. *Journal of Bacteriology*; 191, 3132-3141.

Rico A., & Preston G. M. (2008). *Pseudomonas syringae* pv. *tomato* DC3000 uses constitutive and apoplast-induced nutrient assimilation pathways to catabolize nutrients that are abundant in the tomato apoplast. *Molecular plant-microbe interactions*, 21, 269-282.

Tamir‐Ariel, D., Rosenberg, T., & Burdman, S. (2011). The *Xanthomonas campestris* pv. *vesicatoria* citH gene is expressed early in the infection process of tomato and is positively regulated by the TctDE two‐component regulatory system. *Molecular Plant Pathology,* 12, 57-71.

Shenge, K. C., Mabagala, R. B., & Mortensen, C. N. (2007). Identification and characterization of strains of *Xanthomonas campestris* pv. vesicatoriafrom Tanzania by biolog system and sensitivity to antibiotics. African Journal of Biotechnology, 6(1), 15–22.

Stoyanova, M., Vancheva, T., Moncheva, P. & Bogatzevska, N. (2014). Differentiation of Xanthomonas spp. causing bacterial spot in Bulgaria based on biolog system. *International Journal of Microbiology.* doi: [10.1155/2014/495476](http://dx.doi.org/10.1155/2014/495476).

Vorholt, J. A. (2012). Microbial life in the phyllosphere. *Nature Reviews Microbiology*, *10*(12), 828-840.

Yao, J., & Allen, C. (2006). Chemotaxis is required for virulence and competitive fitness of the bacterial wilt pathogen *Ralstonia solanacearum*. *Journal of bacteriology*, *188*(10), 3697-3708.

Zuluaga A. P., Puigvert M., & Valls M. (2013). Novel plant inputs influencing *Ralstonia solanacearum* during infection. *Frontiers in Microbiology,* 4.

Tang, D. J., He, Y. Q., Feng, J. X., He, B. R., Jiang, B. L., Lu, G. T., & Tang, J. L. (2005). *Xanthomonas campestris* pv. campestris possesses a single gluconeogenic pathway that is required for virulence. *Journal of bacteriology*,*187*(17), 6231-6237.
