# Supplementary material for: Plant and pathogen nutrient acquisition strategies
Source: Front Plant Sci. 2015 Sep 17;6:750. doi: 10.3389/fpls.2015.00750 (PMC4585253; doi:10.3389/fpls.2015.00750)
Supplement: Supplementary file 2 [file Table_2.DOCX]

**Supplementary table S2.** Type of nutrients available in nutritional niches that are colonized by bacterial pathogens in the host plant species^#^.

| SI. No | | Name of different nutrient niche present in plant | †Name of nutrients available at these niches | | | | | | Name of pathogen colonising nutrient niches (examples). | Name of host plant species invaded by pathogens | Reference | |
| --- | --- | --- | --- | --- | --- | --- | --- | --- | --- | --- | --- | --- |
|  |  |  | Sugar | Aminoacid | Organic  acid | Organic alcohol | Mineral | |  |  |  |  |
| 1. | | Phyllosphere | Sucrose  Fructose ʘ  Glucose | All 20 amino acids including GABA; Asn, Gln, Asp, Glu and Ser are major ones | Citrate ʘ  Fumarate  Lactate  Malonate  Malate ʘ | _ | Phosphate  Calcium  Magnesium  Potassium  Nitrogen | | ** ʘ *Pseudomonas syringae* pv*. tomato*  **P.syringae* pv*.syringae* | Tomato  Tobacco | Tukey (1970); Weibull *et al*. (1990); Fiala *et.al* (1990); Leveau and Lindow (2001); Lindow and Brandl (2003); Yu *et al*. (2012); Preiter *et al*. (2005); Mellgren *et al*. (2009); Rico and Preston (2008). | |
| 2. | | Leaf apoplast | Glucose  Fructose  Galctose  Sucrose | All 20 amino acidsincluding GABA; Asp,Ser,Glu  Gln,Pro,Ala,  Val,GABA are major ones | Citrate ʘ  Malonate  Succinate ʘ  Malate ʘ | _ | Calcium  Magnesium  Potassium  Chloride  Sulphate  Phosphate  Nitrate | | * *P. syringae* pv*. tomato*  * ʘ*Xanthomonas campestris* pv*.vesicatoria* | Tomato  Tomato  and Pepper | Ruan *et al*. (1996); Rico, and Preston (2007); Sattelmacher (2000); Zuluaga *et al*. (2013); Vorholt (2012); Tamir-Ariel *et al*. (2007). | |
| 3. | | Phloem | Sucrose  Raffinose  Glucose  Fructose  Galctose | All 20 amino acids including GABA; Amine and acidic amino acid are major ones | Malate  Succinate  Citrate  Malonate  Fumarate | Sorbitol ʘ  Inositol  Mannitol | Potassium  Sodium  Magnesium  Calcium  Phosphate  Nitrate  Chloride | | ***P. syringae* pv*. aesculi*  ***P. syringae* pv*. actinidiae*  **** ʘErwinia amylovora | Horse chestnut  Kiwi fruit  Apple | Aldridge *et al*. (1997); Weibull *et al*. (1990); Fiehn (2002); Green *et al*. (2009); Renzi *et al*. (2012); Vanneste *et al*. (2011); Mullet and webber (2013); (http://plantsinaction.science.uq.edu.au/edition1) | |
| 4. | | Xylem | Glucose  Fructose  Rhammnose  Xylose | Asn  Gln  Ser  Ala  GABA  Glu  Asp | Malate  Citrate  Fumarate  Succinate | Myo-inositol | Potassium, Sodium,  Magnesium, calcium,  Phosphate,  Nitrate,  Chloride,  Sulphate, Molybdeum,  Zinc, copper and iron | | ***ʘ*Ralstonia solanacearum*  *X. albilineans* | Tomato  Sugarcane | Lopez-Millan *et al*. (2000); Iwai *et al*. (2003); Zuluaga *et al*. (2013); Dalsing and Allen (2013); Pieretti *et.al* (2012); (http://plantsinaction.science.uq.edu.au/edition1) | |
| 5. | | Rhizosphere | Fructose  Glucose  Sucrose  Galactose | All 20 amino acid including GABA | Citrate  Fumarate  Malate  Malonate  Succinate | _ | Most of the minerals. | | ******P. syringae* pv*.tabaci* | Tobacco | Dennis *et al*. (2010); Knoche *et al*. (1994). | |
| 6. | | Root apoplast | Glucose  Fructose  Sucrose |  | Malate | _ | Iron  Zinc  Copper  Phosphate | | ∞*Proteobacteria azocarpus* | Rice | Chapleo and Hall (1989); Longnecker *et al*. (1990); Sattelmacher (2000); Reinhold-Hurek *et al*. (2007) | |
|  | | | | | | | | | | | | |
|  | | | | | | |  |  |  |  |  |  |
| ^#^indicates information presented here is based on studies related to pathogen nutrition. † indicates only those nutrients are shown that are related to pathogen. Pathogen invading overlapping nutrient niches are indicated by asterisk; * indicates pathogen overlap in phyllosphere and leaf apoplast; ** indicates pathogen overlap in phyllosphere, phloem and xylem; *** indicates pathogen overlap in Rhizosphere and xylem; **** indicates pathogen overlap in phyllosphere, apoplast, phloem, xylem; ***** indicates pathogen overlap in leaf apoplast and rhizosphere. ∞ indicates non-pathogenic bacteria. ʘ indicates preferred nutrient for particular pathogen shown in same column. | | | | | | | | | | |  |  |

**References:**

Aldridge, P., Metzger, M., & Geider, K. (1997). Genetics of sorbitol metabolism in *Erwinia amylovora* and its influence on bacterial virulence. *Molecular General Genetics*, 256, 611-619.

Chapleo, S. & Hall, J.L. (1989). Sugar Unloading in Roots of Ricinus communis L.: II. Characteristics of Extravascular Apoplast, New Phytologist, 111, 381–390.

Dalsing, B. L., & Allen, C. (2014). Nitrate assimilation contributes to Ralstonia solanacearum root attachment, stem colonization, and virulence. *Journal of bacteriology*, *196*(5), 949-960.

Dennis, P. G., Miller, A. J., Hirsch, P. R. (2010). Are root exudates more important than other sources of rhizodeposits in structuring rhizosphere bacterial communities?. *FEMS microbiology ecology*, 72, 313-327.

Fiala, V., Glad, C., Martin, M., Jolivet, E., & Derridj, S. (1990). Occurrence of soluble carbohydrates on the phylloplane of maize (*Zea mays* L.): variations in relation to leaf heterogeneity and position on the plant. *New Phytologist*, 115, 609-615.

Fiehn, O. (2003). Metabolic networks of *Cucurbita maxima* phloem. *Phytochemistry*, 62, 875-886.

Green, S., Laue, B., Fossdal, C. G., A’Hara, S. W., & Cottrell, J. E. (2009). Infection of horse chestnut (Aesculus hippocastanum) by Pseudomonas syringae pv. aesculi and its detection by quantitative real‐time PCR. *Plant Pathology*, *58*(4), 731-744.

Iwai, H., Usui, M., Hoshino, H., Kamada, H., Matsunaga, T., Kakegawa, K., & Satoh, S. (2003). Analysis of sugars in squash xylem sap. *Plant and cell physiology*, *44*(6), 582-587.

Knoche, K. K., Parke, J. L., & Durbin, R. D. (1994). Relationship of Pseudomonas syringae pv. tabaci races to the rhizosphere of Wisconsin-grown tobacco. *Plant and soil*, *158*(1), 91-97.

Leveau J. H., & Lindow S. E. (2001). Appetite of an epiphyte: quantitative monitoring of bacterial sugar consumption in the phyllosphere. *Proceedings of the National Academy of Sciences*, 98, 3446-3453.

Lindow, S. E., & Brandl, M. T. (2003). Microbiology of the phyllosphere. *Applied and environmental microbiology*, 69, 1875-1883.

Longnecker, N., & Welch, R. M. (1990). Accumulation of Apoplastic Iron in Plant Roots A Factor in the Resistance of Soybeans to Iron-Deficiency Induced Chlorosis?. *Plant physiology*, *92*(1), 17-22.

López-Millán, A. F., Morales, F., Abadı́a, A., & Abadı́a, J. (2000). Effects of iron deficiency on the composition of the leaf apoplastic fluid and xylem sap in sugar beet. Implications for iron and carbon transport. *Plant Physiology*, *124*(2), 873-884.

Mellgren E. M., Kloek A. P., & Kunkel B. N. (2009). Mqo, a tricarboxylic acid cycle enzyme, is required for virulence of *Pseudomonas syringae* pv. *tomato* strain DC3000 on *Arabidopsis thaliana*. *Journal of Bacteriology*; 191, 3132-3141.

Mullett, M. S., & Webber, J. F. (2013). Pseudomonas syringae pv. aesculi: foliar infection of Aesculus species and temperature–growth relationships.*Forest Pathology*, *43*(5), 371-378.

Pieretti, I., Royer, M., Barbe, V., Carrere, S., Koebnik, R., & Couloux, A. (2012). Genomic insights into strategies used by *Xanthomonas albilineans* with its reduced artillery to spread within sugarcane xylem vessels. *BMC Genomics,* 13, 658.

Reinhold-Hurek, B., & Hurek, T. (2011). Living inside plants: bacterial endophytes. *Current opinion in plant biology*, *14*(4), 435-443.

Rico A., & Preston G. M. (2008). *Pseudomonas syringae* pv. *tomato* DC3000 uses constitutive and apoplast-induced nutrient assimilation pathways to catabolize nutrients that are abundant in the tomato apoplast. *Molecular plant-microbe interactions*, 21, 269-282.

Ruan, Y. L., Patrick, J. W., & Brady, C. J. (1996). The composition of apoplast fluid recovered from intact developing tomato fruit. Functional Plant Biology,23(1), 9-13.

Sattelmacher, B. (2001). The apoplast and its significance for plant mineral nutrition. *New Phytologist*, 149, 167-192.

Tukey, Jr H. B. (1970). The leaching of substances from plants. *Annual review of plant physiology*, 21, 305-324.

Vanneste, J.L., Yu, J., Cornish, D.A., Max, S. & Clark, G. (2011). Presence of Pseudomonas syringae pv. actinidiae, the causal agent of bacterial canker of kiwifruit on symptomatic and asymptomatic tissues of kiwifruit. *N. Z. Plant Protect.* 64, 241–245.

Weibull, J., Ronquist, F., & Brishammar, S. (1990). Free Amino Acid Composition of Leaf Exudates and Phloem Sap A Comparative Study in Oats and Barley. *Plant Physiology*, 92, 222-226.

Yu, X., Lund, S. P., Scott, R. A., Greenwald, J. W., Records, A. H., Nettleton, D.,Lindow, S. E., Gross, D. C., & Beattie, G. A. (2013). Transcriptional responses of Pseudomonas syringae to growth in epiphytic versus apoplastic leaf sites. *Proceedings of the National Academy of Sciences*, 110, e425-e434.

Zuluaga A. P., Puigvert M., & Valls M. (2013). Novel plant inputs influencing *Ralstonia solanacearum* during infection. *Frontiers in Microbiology,* 4.
