# Supplementary material for: Plant and pathogen nutrient acquisition strategies
Source: Front Plant Sci. 2015 Sep 17;6:750. doi: 10.3389/fpls.2015.00750 (PMC4585253; doi:10.3389/fpls.2015.00750)
Supplement: Supplementary file 3 [file Table_3.DOCX]

**Supplementary table S3.** Composition of different nutrient niches in plant species*.

| SI.No. | Nutrients | Sugar beet  (*Beta vulgaris*) | | Acorn Banksia (*Banksia prionotes*) | | Squash  (*Cucurbita maxima*) | | Tomato  (*Solanum lycopersicon*) | | |
| --- | --- | --- | --- | --- | --- | --- | --- | --- | --- | --- |
|  |  | Leaf apoplast (mM) | Xylem (mM) | Xylem (mM) | Phloem (mM) | Phloem (mM) | Xylem (mM) | Leaf apoplast (mM) | fruit apoplast (mM) | Xylem (mM) |
| 1 | Sugar  Sucrose  Glucose  Fructose  Galactose | 2.6  16.4  4.5  - | Ab  20  27.5  - | Ab  -  -  - | 493  -  -  - | 0.8  2  2.7  - | -  132.7(23.9)  -  115(20.7) | 565.5(193.43)  616.2(110.93)  870.7(156.74)  81.5(14.67) | -  13.4± 2.3  18.2± 2.5  - | -  22(4.08)  47(8.54)  0.0 |
| 2 | Sugar alcohol  Inositol  Myo-inositol | -  - | -  - | -  - | -  - | 0.4  - | -  84.4(15.2) | -  - | -  - | -  - |
| 3 | Organic acid  Citrate  Malate  Fumarate  Succinate  Malonate | 0.2  2.1  -  3.5  - | 0.7  0.7  -  1.4  - | -  0.42  -  -  - | -  4.28  -  -  - | 2.1  1.6  0.21  0.18  - | -  -  -  -  - | -  -  -  -  - | 3.4± 0.2  2.1 ±0.2  -  -  - | -  -  -  -  - |
| 4 | Aminoacid  GABA  Aspartic acid  Glutamic acid  Alanine  Serine  Glutamine  Leucine  Glycine  Phenylalanine  Methionine  Valine  Lysine  Threonine  Isoleucine  Tyrosine  Proline  Asparagine  Histidine  Arginine  Cysteine  Tryptophan | 0.056±.011  0.262±0.02  0.320±0.003  0.303±0.130  0.121±0.042  0.450±0.180  <0.001  0.056±0.008  -  -  0.060±0.055  0.031±0.0005  0.044±0.015  0.016±0.016  -  <0.001  0.059±0.010  <0.001  -  -  - | -  -  -  -  -  -  -  -  -  -  -  -  -  -  -  -  -  -  -  -  - | 0.53  -  -  -  -  -  -  -  -  -  -  -  -  -  -  -  -  -  -  -  -  - | 2.35  -  -  -  -  -  -  -  -  -  -  -  -  -  -  -  -  -  -  -  -  - | 0.8  6.5  6.0  0.3  11.4  0.36  0.09  0.25  0.26  0.19  0.03  2.1  0.17  0.13  -  -  -  -  0.34  -  - | -  -  -  -  -  -  -  -  -  -  -  -  -  -  -  -  -  -  -  -  - | 0.5994 (0.5755± 0.0829)  0.2811 (0.1454 ± 0.0455)  0.2743 (0.1374 ± 0.0450)  0.1567 (0.0673 ± 0.0139)  0.0194 (0.0512 ± 0.0123)  0.1469 (0.0340 ± 0.0094)  0.0856 (0.0318 ± 0.0570)  - (0.0288 ± 0.0097)  0.0489 (0.0252 ± 0.0026)  - (0.0249 ± 0.0086)  0.0835 (0.0247 ± 0.0001)  0.0539(0.0225 ± 0.002)  - (0.0220 ± 0.0089)  0.0465 (0.0194 ± 0.0042)  0.0210 (0.0160 ± 0.0026) 0.0763 (0.0128 ± 0.0017)  0.0642 (0.0095 ± 0.0027)  0.0190 (0.0068 ± 0.002)  0.0270 ( - )  -  - | 4.1 ±0.5  0.6 ±0.1  0.6± 0.2  0.6 ±0.0  5.9 ±0.6  -  -  -  0.3 ±0.2  0.5 ±0.0  -  -  -  1.4± 0.1  -  -  -  -  -  -  - | 0.0622  0.0132  0.0113  0.0017  0.0055  0.1955  0.0247  -  0.0050  -  0.0517  0.0494  -  0.0248  0.0068  0.0041  0.0940  0.0250  0.0282  -  - |
| 5 | Minerals  Potassium  Sodium Magnesium | 30  -  2 | 80-130  -  4.9 | 2.39  1.84  0.55 | 15.2  24.1  6.36 | -  -  - | -  -  - | -  -  - | 19.1± 2.5  0.5± 0.1  5.0 ±06 | -  -  - |
|  | References | Lopez-Millan *et al*. (2000) | | <http://plantsinaction>. science.uq.edu.au/ edition1 | | Fiehn (2003) | Iwai *et al*. (2003) | Ruan *et al*. (1996) ; Zuluaga *et al*. (2013) ; values in bracket taken from Rico and Preston (2007) | | |

Asterisk (*) indicate that it is merely the compilation of data from different research articles. Soft hyphen (-) indicate information were not available for particular nutrient in the literature. Absent (Ab) indicate nutrient is not present in particular nutrient niche.

**References:**

Fiehn, O. (2003). Metabolic networks of *Cucurbita maxima* phloem. *Phytochemistry*, 62, 875-886

Iwai, H., Usui, M., Hoshino, H., Kamada, H., Matsunaga, T., Kakegawa, K., & Satoh, S. (2003). Analysis of sugars in squash xylem sap. *Plant and cell physiology*, *44*(6), 582-587.

López-Millán, A. F., Morales, F., Abadı́a, A., & Abadı́a, J. (2000). Effects of iron deficiency on the composition of the leaf apoplastic fluid and xylem sap in sugar beet. Implications for iron and carbon transport. *Plant Physiology*, *124*(2), 873-884.

Renzi, M., Copini, P., Taddei, A. R., Rossetti, A., Gallipoli, L., & Mazzaglia, A. (2012). Bacterial canker on kiwifruit in Italy: anatomical changes in the wood and in the primary infection sites. *Phytopathology*; 102, 827-840.

Rico A., & Preston G. M. (2008). *Pseudomonas syringae* pv. *tomato* DC3000 uses constitutive and apoplast-induced nutrient assimilation pathways to catabolize nutrients that are abundant in the tomato apoplast. *Molecular plant-microbe interactions*, 21, 269-282.

Ruan, Y. L., Patrick, J. W., & Brady, C. J. (1996). The composition of apoplast fluid recovered from intact developing tomato fruit. Functional Plant Biology,23(1), 9-13.

Tamir‐Ariel, D., Rosenberg, T., & Burdman, S. (2011). The *Xanthomonas campestris* pv. *vesicatoria* citH gene is expressed early in the infection process of tomato and is positively regulated by the TctDE two‐component regulatory system. *Molecular Plant Pathology,* 12, 57-71.

Vorholt, J. A. (2012). Microbial life in the phyllosphere. *Nature Reviews Microbiology*, *10*(12), 828-840.

Weibull, J., Ronquist, F., & Brishammar, S. (1990). Free Amino Acid Composition of Leaf Exudates and Phloem Sap A Comparative Study in Oats and Barley. *Plant Physiology*, 92, 222-226.

Zuluaga A. P., Puigvert M., & Valls M. (2013). Novel plant inputs influencing Ralstonia solanacearum during infection. Frontiers in Microbiology, 4.
