# Supplementary material for: Plant and pathogen nutrient acquisition strategies
Source: Front Plant Sci. 2015 Sep 17;6:750. doi: 10.3389/fpls.2015.00750 (PMC4585253; doi:10.3389/fpls.2015.00750)
Supplement: Supplementary file 4 [file Table_4.DOCX]

**Supplementary table S4**. Bacterial effector molecules involved in plant nutrient manipulation

| Sl. No. | Name of the plant species | Name of the host pathogen | Name of effector molecules deployed by pathogen in column 3 for nutrient pathway manipulation | Plant nutrient machinery and its mechanism targeted by the effector | Name of nutrient pathway manipulated | Name of nonhost pathogen | State of nonhost pathogen interaction | References |
| --- | --- | --- | --- | --- | --- | --- | --- | --- |
| 1 | Rice (*Oryza sativa*) | *Xanthomonas oryzae* pv.*oryzae* PXO99 | PthXo 1 | Upregulation of SWEET11 transporter | Efflux of glucose | *X. campestris* pv *holcicola* (host pathogen of maize) | Absence of PthXo1, TAL effector is the reason attributed for nonhost pathogen inability to infect rice | Chen *et al*. 2010 |
| 2 | Rice | *X. oryzae* pv. *oryzae* PXO99 | AvrXa7 | Upregulation of SWEET14 transporter | Efflux of glucose | *X. campestris* pv *holcicola* (host pathogen of maize) | Absence of AvrXa7, TAL effector is the reason attributed for nonhost pathogen inability to infect rice | Chen *et al*. 2010 |
| 3 | Pepper (*Piper nigrum*) | *X .campestris* pv. *vesicatoria* | XopB | Suppression of cellwall-bound invertase. Pre-vent initiation of sugar mediated defense signalling | Prevent generation of hexoses | *X. axonopodis* pv. *glycines* (host pathogen of soya bean) | Absence of XopB is the reason attributed for nonhost pathogen inability to infect pepper | Sophia Sonnewald *et al*. 2012 |

**References:**

Chen, L. Q., Hou, B. H., Lalonde, S., Takanaga, H., Hartung, M. L., Qu, X. Q., Guo, W. J., & Frommer, W. B. (2010). Sugar transporters for intercellular exchange and nutrition of pathogens. *Nature*, 468, 527-532.

Sonnewald, S., Priller, J. P., Schuster, J., Glickmann, E., Hajirezaei, M. R., & Siebig, S. (2012). Regulation of cell wall-bound invertase in pepper leaves by *Xanthomonas campestris* pv. *vesicatoria* type three effectors. *PloS one*, 7, e51763.
