# Supplementary material for: Plant and pathogen nutrient acquisition strategies
Source: Front Plant Sci. 2015 Sep 17;6:750. doi: 10.3389/fpls.2015.00750 (PMC4585253; doi:10.3389/fpls.2015.00750)
Supplement: Supplementary file 5 [file Table_5.DOCX]

**Supplementary table S5.** List of plant genes attributed for their involvement in imparting nonhost resistance by restricting the availability of nutrients to bacterial pathogens.

| Sl. No | Plant species | Gene name | Function of gene | Molecular mechanism | Role in plant innate immunity | References |
| --- | --- | --- | --- | --- | --- | --- |
| 1 | *Nicotiana benthamiana* | *NbSQS* | Gene encoding squalene synthase, catalyzing the first step in biosynthesis of phytosterol | Phytosterol level help in maintaining the membrane permeability characteristics and thereby regulating nutrient release from cytosol into apoplast | *NbSQS*-silenced plants fully compromised nonhost resistance and partially compromised basal resistance | Wang *et al*. (2012) |
| 2 | *Arabidopsis thaliana* | *AtSMT-2* | Gene encoding sterol methyl transferase 2,a key branching point enzyme catalysing second methyl transfer reaction leading to biosynthesis of β-sitosterol and stigmasterol | Phytosterol level help in maintaining the membrane permeability characteristics and thereby regulating nutrient release from cytosol into apoplast | *Atsmt2* mutant compromised host and nonhost resistance | Wang *et al*. (2012) |
| 3 | *Arabidopsis thaliana* | *AtCYP710A1* | Gene encoding C22-sterol desaturase that convert β-sitosterol to stigmasitosterol | Stigmasterol level plays role in maintaining membrane integrity and there by controlling nutrient release from cytosol into apoplast | *Atcyp710a1* mutant compromised host and nonhost resistance | Wang *et al*. (2012) |

**Reference:**

Wang, K., Senthil-Kumar, M., Ryu, C. M., Kang, L., & Mysore, K. S. (2012). Phytosterols play a key role in plant innate immunity against bacterial pathogens by regulating nutrient efflux into the apoplast. *Plant physiology*, 158, 1789-1802.
